# Administration of Bleomycin via the Oropharyngeal Aspiration Route Leads to Sustained Lung Fibrosis in Mice and Rats as Quantified by UTE-MRI and Histology

**DOI:** 10.1371/journal.pone.0063432

**Published:** 2013-05-07

**Authors:** Christine Egger, Catherine Cannet, Christelle Gérard, Elizabeth Jarman, Gabor Jarai, Agnès Feige, Thomas Suply, Arthur Micard, Andrew Dunbar, Bruno Tigani, Nicolau Beckmann

**Affiliations:** 1 Global Imaging Group, Novartis Institutes for BioMedical Research, Basel, Switzerland; 2 Biocenter, University of Basel, Basel, Switzerland; 3 Respiratory Diseases Department, Novartis Institutes for BioMedical Research, Horsham, United Kingdom; 4 Developmental and Molecular Pathways Department, Novartis Institutes for BioMedical Research, Basel, Switzerland; Helmholtz Zentrum München/Ludwig-Maximilians-University Munich, Germany

## Abstract

Pulmonary fibrosis can be experimentally induced in small rodents by bleomycin. The antibiotic is usually administered via the intratracheal or intranasal routes. In the present study, we investigated the oropharyngeal aspiration of bleomycin as an alternative route for the induction of lung fibrosis in rats and mice. The development of lung injury was followed *in vivo* by ultrashort echo time magnetic resonance imaging (UTE-MRI) and by post-mortem analyses (histology of collagen, hydroxyproline determination, and qRT-PCR). In C57BL/6 mice, oropharyngeal aspiration of bleomycin led to more prominent lung fibrosis as compared to intranasal administration. Consequently, the oropharyngeal aspiration route allowed a dose reduction of bleomycin and, therewith, a model refinement. Moreover, the distribution of collagen after oropharyngeal aspiration of bleomycin was more homogenous than after intranasal administration: for the oropharyngeal aspiration route, fibrotic areas appeared all over the lung lobes, while for the intranasal route fibrotic lesions appeared mainly around the largest superior airways. Thus, oropharyngeal aspiration of bleomycin induced morphological changes that were more comparable to the human disease than the intranasal administration route did. Oropharyngeal aspiration of bleomycin led to a homogeneous fibrotic injury also in rat lungs. The present data suggest oropharyngeal aspiration of bleomycin as a less invasive means to induce homogeneous and sustained fibrosis in the lungs of mice and rats.

## Introduction

Pulmonary fibrosis, characterized by fibroblast proliferation and extracellular matrix remodeling, is the end result of various types of lung damage including interstitial pneumonia and respiratory bronchiolitis [1]–[3]. A patchy alveolar wall fibrosis develops in patients with chronic obstructive pulmonary disease (COPD) whereas in chronic asthmatics, a fibrotic response occurs predominantly in the lamina reticularis, leading to thickening of the basement membrane [4], [5]. In both cases the ongoing inflammation-repair cycle leads to permanent structural changes in the airway wall (remodeling) of which fibrosis is a major constituent [6], [7]. However, up to 50% of the cases of pulmonary fibrosis are defined as idiopathic pulmonary fibrosis (IPF) [8].

Bleomycin is an antibiotic with activity against gram-negative bacteria [9] that possesses chemotherapeutic properties and is highly efficient in some types of carcinomas. In spite of these therapeutic characteristics, bleomycin may produce a dose-dependent pulmonary fibrosis in a significant portion of patients [8]. Therefore, single [10]–[12] or multiple [13] instillation of bleomycin is commonly used to induce experimental pulmonary fibrosis in small rodents. Application of the antibiotic causes an acute inflammatory reaction and fibrotic changes that resemble human fibrotic lung disease both histologically and physiologically [14], [15]. The availability of this animal model of pulmonary fibrosis provides the opportunity to investigate novel pharmacological approaches aiming to treat this crippling disease.

The intra-tracheal (IT) route of administration has been used to deliver bleomycin into the lungs of rats and mice [13], [16]–[18]. Additionally, the intra-nasal (IN) route has also been adopted in mice. Arguments for favoring the IN administration protocol in this species are the method’s simplicity and speed, and that it does not injure the trachea. A major disadvantage of the IN administration route is the inhomogeneous distribution of the liquid in the respiratory tract, which is dependent on different parameters as the volume of instilled fluid and the depth of anaesthesia [19].

An alternative technique called oropharyngeal aspiration (OA) has been developed to administer substances into the lungs of mice. One of the first applications of this method can be found in a study by Foster et al., who used OA for the instillation of ^99m^TC-labelled sulfur colloid to measure mucociliary function in mice by scintigraphy [20]. Rao et al. used the technique to administer fluorescent amine-modified polystyrene latex beads and beryllium oxide particles into the mouse lung. They found the technique to be simple and reproducible, and the exposures of the lung to be highly correlated to the administered doses of substrates [21]. A comparison of IT to OA administration was performed by Lakatos et al. in a mouse model of silicosis [22]. Mice treated with crystalline silica administered via the OA route developed fibrosis with a more homogenous distribution all over the lung and with less variability. The OA method has also been used in other studies as for instance a mouse model of chemical-induced asthma [23] or a murine model of diacetyl toxicity [24].

Recently we reported the use of magnetic resonance imaging (MRI) to non-invasively follow the course of lung injury induced by repeated IN administration of bleomycin to mice [25], or of single or repeated IT bleomycin administration to rats [17]. In the present work this model of lung fibrosis has been further refined by adopting the OA administration route for bleomycin. The responses in the lungs detected in vivo by MRI have been compared to histology, hydroxyproline determination and quantitative real-time polymerase chain reaction (qRT-PCR) analyses.

## Materials and Methods

### Ethics Statement

Experiments were carried out with the approval of the Veterinary Authority of the City of Basel (license number BS-1989).

### Animals

Seven- to 9-week-old C57BL/6 (n = 35) or BALB/c (n = 37) male mice or Sprague Dawley rats (n = 10) (Elevage Janvier, Saint Berthevin, France) were used throughout the study. Animals were kept at an ambient temperature of 22±2°C under a 12 h normal phase light-dark cycle and fed NAFAG® pellets (Nahr- und Futtermittel AG, Gossau, Switzerland). Drinking water and food were freely available.

### Intra-nasal Administration of Bleomycin or Saline

**Figure 1:**
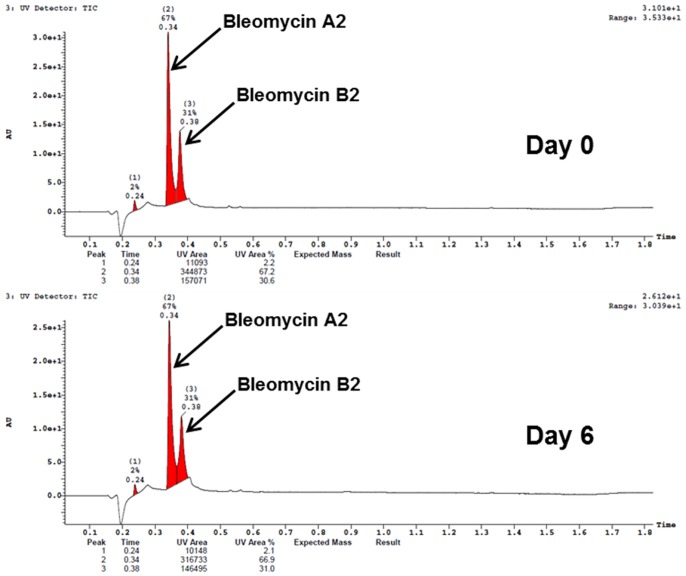
HPLC/MS spectra of bleomycin (0.075 mg/mL) freshly prepared and after six days at 4°C acquired on an Acquity Ultra performance LC instrument (Waters, Milford, Massachusetts, USA). An Acquity HSS T3 1.8 µm 2.1×50 mm column was used at 60°C; eluent A (water +0.05% formic acid +3.75 mM ammonium acetate) and eluent B (acetonitrile +0.04% formic acid) were used with a gradient of 5 to 98% B in 1.4 min and 1.0 mL/min flow. Bleomycin A2: C55H84N17O21S3+ with a mass of 1415.56 g/mol; bleomycin B2: C55H84N20O21S2 with a mass of 1425.52 g/mol.

Mice were lightly anesthetized with 2.0% isoflurane (Abbott, Cham, Switzerland) delivered in a box and bleomycin hydrochloride (Teva, Basel, Switzerland), 0.25 mg/kg in 25 µl of saline (0.9%) or vehicle (25 µl of saline (0.9%)) was administered IN with a micropipette (12.5 µl per nostril). Animals were allowed to recover immediately afterwards. This procedure was performed six times consecutively, once daily. Bleomycin solution was prepared before the first administration and then stored, in aliquots, at 4°C for administration at the further timepoints. A similar administration protocol has been shown earlier to induce substantial and sustained pulmonary fibrosis in mice [25]. The bleomycin solution kept at 4°C was stable over 6 days as verified by high performance liquid chromatography/mass spectrometry (HPLC/MS) analyses (Figure 1), a result that is consistent with earlier studies demonstrating that bleomycin in solution at room temperature is stable for more than two weeks [26].

### Oropharyngeal Aspiration of Bleomycin or Saline in Mice

Animals were lightly anesthetized with 2% isoflurane delivered in a box and bleomycin hydrochloride (0.1 or 0.25 mg/kg for C57BL/6 mice; 0.5 or 1 mg/kg for BALB/c mice) in 40 µl of saline (0.9%) or vehicle (40 µl of saline (0.9%)) was administered via OA using a micropipette. OA was performed as described by de Vooght et al. [23]. Mice were fixed on a surgery board, the tongue was pulled out with a forceps, and the liquid was placed onto the distal part of the oropharynx while the nose was gently closed. As for the intra-nasal administration protocol, the procedure was performed six times consecutively, once daily.

### Oropharyngeal Aspiration of Bleomycin in Rats

Animals were lightly anesthetized with 3.5% isoflurane delivered in a box and bleomycin hydrochloride, 2 mg/kg in 100 µl of saline (0.9%) was administered via OA with a micropipette using a similar procedure as described above for mice.

### Magnetic Resonance Imaging (MRI)

During MRI signal acquisitions, animals were placed in supine position in a cradle made of Plexiglas. Body temperature was kept at 37±1°C using warm air. Following a short period of introduction in a box, anesthesia was maintained with 1.5% isoflurane for mice and 2% for rats in a mixture of O_2_/N_2_O (1∶2), administered via a nose cone. All measurements were performed on spontaneously breathing animals; neither cardiac nor respiratory triggering was applied. As demonstrated earlier [25], [27], averaging over several respiratory cycles suppressed artifacts caused by movements of the chest and the heart without the necessity of triggering the data acquisition. Measurements were carried out with a Biospec 47/40 spectrometer (Bruker Medical Systems, Ettlingen, Germany) operating at 4.7 T and equipped with an actively shielded gradient system capable of generating a gradient of 200 mT/m. The operational software of the scanner was Paravision (Bruker).

An ultra-short echo time (UTE) sequence [28]–[30] with the following parameters was applied for the detection of bleomycin-induced lung injury in mice: repetition time 20.0 ms, echo time 529 µs, 604 projections, 2 averages, band width 200 kHz, flip angle of the excitation pulse 25°, matrix size 192×192, slice thickness 1.4 mm and field of view 3.0 × 3.0 cm^2^. The total acquisition time was of 4.0 min for 10 consecutive axial slices covering the entire lung. In an initial assessment, different numbers of averages were tested: 1 average (2-min-acquisition-time), 2 averages (4-min-acquisition-time), 4 averages (8-min-acquisition-time), and 8 averages (16-min-acquisition-time). It was found that the acquisition with 2 averages was appropriate and then used throughout the study. A birdcage resonator of 32 mm diameter was used for excitation and detection. For rats an UTE sequence with the following parameters was used: repetition time 8.0 ms, echo time 528 µs, 604 projections, 5 averages, band width 200 kHz, flip angle of the excitation pulse 25°, matrix size 192×192, slice thickness 2.0 mm and field of view 6.0 × 6.0 cm^2^. The total acquisition time was of 7.25 min for 18 consecutive transverse slices covering the entire lung. A birdcage resonator of 70 mm diameter was used for excitation and detection.

### MR Image Analysis

**Figure 2:**
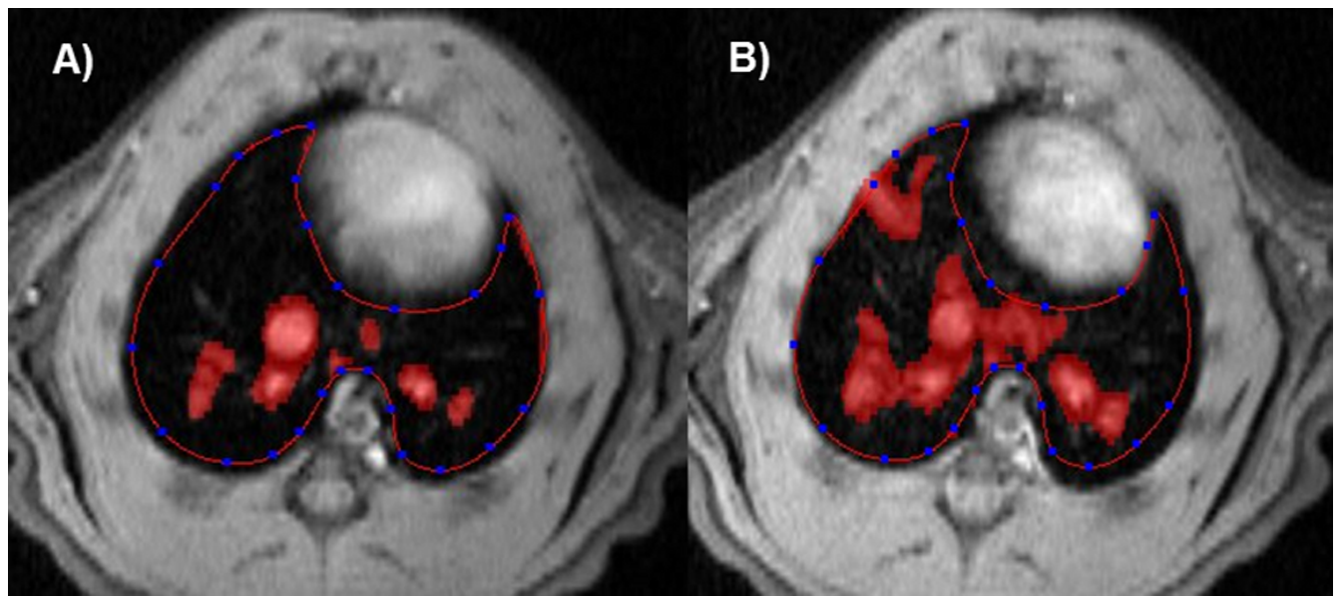
Details of segmentation procedure. For each slice, a border is drawn manually to limit the segmentation of high intensity signals to the lung. Images correspond to a rat at A) baseline and B) day 7 after OA of bleomycin (2 mg/kg).

At a given time point, the area of bleomycin-induced lesions was quantified on each image from the dataset covering the whole lung, using a semi-automatic segmentation procedure implemented in the IDL (Interactive Data Language Research Systems, Boulder, Colorado, USA) environment on a Linux system. Images were first lowpass-filtered with a Gaussian profile filter and then transformed into a set of four grey level classes using adaptive Lloyd-Max histogram quantitation. The highest class in the transformed images was extracted interactively by a region grower, whose border was drawn manually on each slice to control the growing and limit it to areas within the lung (Figure 2). For each image, the area thus segmented by region growing corresponded to high intensity signals in the lung. The total volume of high intensity signals was then calculated by adding the areas obtained for each image from the dataset, and multiplying the summed value by the slice thickness. Segmentation parameters were the same for all analyzed images.

### Post-mortem Analyses

Animals were sacrificed with an overdose of thiopental (Pentothal®, Abbott; 250 mg/kg i.p., 0.2 ml) immediately after an MRI acquisition. The trachea was immediately ligated to avoid collapse of the lung. Following the organ’s removal, the left lobe was used for histological analyses, while the right lobes were employed for hydroxyproline and qRT-PCR analyses.

### Histology

**Figure 3:**
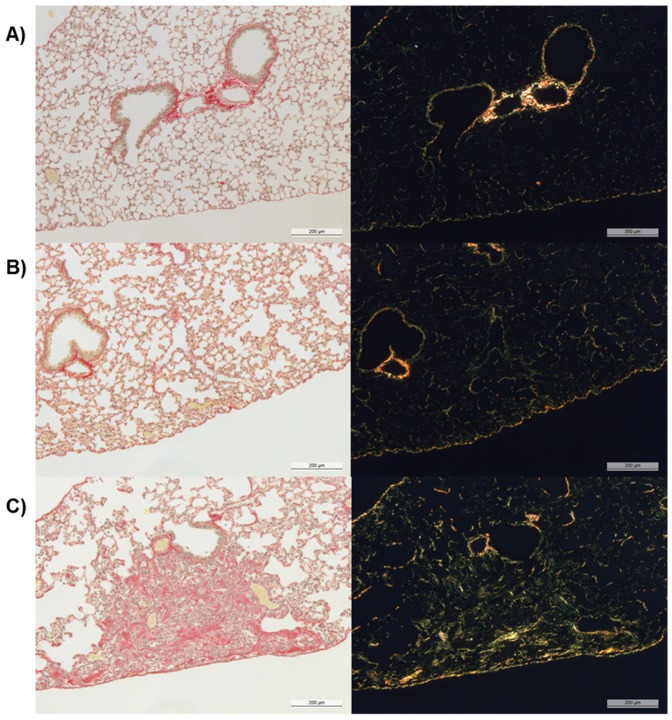
Picrosirius-stained histological slices of C57BL/6 mice observed at bright-field (left column) and at polarization (right column). The high intensity areas in the polarized view, corresponding to collagen, coincide well with the red areas in the bright-field view. In this work, collagen has been quantified by examining histological slices at bright-field. A) Saline-challenged mouse (OA administration). B) IN administration of bleomycin (6×0.25 mg/kg). C) OA administration of bleomycin (6×0.25 mg/kg).

Histological analysis was performed as described in more detail earlier [27]. Lung lobes were immersed in 10% neutral buffered formalin for 24 h. Following fixation, lungs were trimmed, and three transverse sections approximately 0.8 mm thick were cut through the left lung (superior, median, and caudal parts) to include the main bronchi as well as the pulmonary alveoli. Sections were then dehydrated through increasing graded series of ethylic alcohol and embedded in one block of paraffin wax. Three serial histological slices (3 µm) were obtained from each section and stained with picrosirius red for the identification of collagen fibers and newly synthesized collagen (Figure 3). The age of fibrotic lesions can be assessed quantitatively in picrosirius red stained slices using polarized light microscopy [31]–[37]. Collagen birefringence increases as the tissue ages. Such changes in collagen birefringence manifest as changes in brightness of the polarization image and the polarization brightness of a fibrotic network is a useful parameter to characterize the maturity of a scar. In the present work, the aim was not to date fibrotic lesions but to detect and quantify collagen content. Thus, picrosirius stained histological slices have been examined under bright field illumination. The quantification of collagen in picrosirius stained slices using bright field has been described elsewhere [38]–[44]. For each animal, a mean value was derived from the analysis of 9 histological slices.

Collagen was quantified using the program “Histolab” (Microvision Instruments, Evry, France). Picrosirius-stained slides were examined with a light microscope (Eclipse E600, Nikon, Egg, Switzerland) connected to a CCD progressive scan video color camera (XCD-U100 CR, Sony, Tokyo, Japan). The whole surface of three slices of the left lung was captured at ×10 magnification. The color corresponding to picrosirius was extracted by threshold setting and the area corresponding to picrosirius staining calculated. The percentage of picrosirius to the total lung surface analyzed was calculated for each animal. Results were shown as picrosirius factors, expressing the collagen content in the lungs of bleomycin-challenged animals relative to the mean collagen content in the lungs of saline-treated animals.

### Determination of Hydroxyproline in Lung Tissue Samples

Lung tissue samples were weighed, dried in an oven at 90°C overnight, and the weight was recorded again. Dry tissues were then boiled in 0.5 mL of 6 M HCl at 120°C overnight (8–16 hours) in Pyrex tubes with heat- and acid-resistant screw-on caps (article TES-830-70G; Fisher Scientific, Wohlen, Switzerland). After cooling down and adding 5 µL of phenolphthalein (1%), the samples were neutralized with NaOH 10 M (Sigma N°S-5881) and 6 M HCl. Black precipitate and brown colour was removed by adding 100 µL of carbon suspension [10 mg/mL acitvated charcoal (article C4386; Sigma-Aldrich, Buchs, Switzerland) in water], centrifugation, and filtration. Five microliters of standard or hydrolysed sample were pipetted in triplicate onto a 96 well plate, and 5 µL citrate acetate buffer (5% citric acid, 7.2% sodium acetate, 3.4% sodium hydroxide, 1.2% glacial acetic acid, distilled water) was added to each well, as well as 100 µL of freshly prepared chloramine-T solution (14.1 mg chloramine-T, 0.1 mL n-propanol, 0.1 mL distilled water, 0.8 mL citrate acetate buffer). The samples were then incubated at room temprature for 20 minutes. After adding 100 µL of Ehrlich’s reagent (2.5 g of 4-(dimethylamino)benzaldehyde, 9.3 mL of n-propanol, and 3.9 mL of 70% perchloric acid), the wells were incubated for 20 minutes at 65°C. After cooling down, the samples were measured at 550 nm on a spectrophotometer (SpectraMax 340PC, Molecular Devices, Sunnyvale, CA, USA), a standard curve from 5 to 100 mg hydroxyproline in water was created. Data were expressed as µg/right lung lobe.

### Quantitative Real-time Polymerase Chain Reaction (qRT-PCR)

Removed lung tissue was stabilized in liquid nitrogen and stored at −80°C until use. For RNA purification, samples were transferred to a 2 ml Eppendorf tube with 1000 µl of RLT buffer (Qiagen, Hombrechtikon, Switzerland; #79216) and β-mercaptoethanol (Sigma-Aldrich; #M3148) and one stainless bead (Qiagen, #69989). Samples were homogenized (Qiagen Tissue Lyser, #85300) in two to three 1-min runs at 30 rotations/s each, with 1 min on ice between the runs. After centrifugation for 3 min at 13 000 rpm, 4°C, 300 µl of the resulting supernatant were processed on the Rneasy Mini Kit (Qiagen, #74106) with a DNAse digestion step (Qiagen, #79254) following the manufacturer’s protocol. Resulting total RNAs were quantified on a NanoDrop™ system (NanoDrop Technologies Inc., Wilmington, DE, USA) and 1 µg was reverse-transcribed using the high capacity cDNA RT kit (Applied Biosystems, Zug, Switzerland; #4368813).

Expression of the different genes of interest was evaluated by real time PCR using the ABI Prism 7900HT system (Applied Biosystems). Briefly, 10 ng/µl equivalent RNA per well was distributed in a 384 well plates (Applied Biosystems, #4326270), with 5 µl of the Taqman Universal Mastermix 2× kit (Applied Biosystems, #4324020) and 0.5 µl of 20× Assay-on-demand mix Taqman probe (Applied Biosystem, #4331182). The probes used were Mm00801666_g1 (Collagen type I, alpha 1; Col1α1), Mm00802529_m1 (epidermal growth factor-like module containing mucin-like, hormone receptor-like sequence 1, F4/80), and Mm00500554_m1 (matrix metalloproteinase 12; MMP12). Expression for each sample was normalized to HPRT (probe Mm03024075 m1) and compared to the vehicle-treated group, using the 2^−ΔΔCT^ formula (2^−(ΔCT1−ΔCT2)^), where ΔCT1 is the averaged CT value of a sample, normalized to HPRT and ΔCT2 is the average of ΔCT in the control group (vehicle group), also normalized to the housekeeping gene, resulting in the relative fold induction. The expression of HPRT was the same in all analyzed groups.

### Near-infrared-fluorescence (NIRF) Imaging for Distribution Analyses

For comparison of the administration routes, in a separate set of experiments isoflurane-anesthetized mice received a solution containing the fluorescent dye Cy5.5 either IN (25 µL; 12.5 µL per nostril) or OA (25 µL). Animals were allowed to recover from anesthesia immediately after solution administration. However, they were sacrificed 60 min later with an overdose of thiopental (250 mg/kg i.p., 0.2 mL). The trachea was immediately ligated to avoid collapse of the lungs, which were harvested from the body. NIRF imaging was performed on the isolated lungs using a Photon Imager (Biospace Lab, Paris, France). For fluorescence excitation, a Xenon lamp at 660 nm was used. The fluorescent light emitted from the sample was detected by a charge-coupled device camera, equipped with a focusing lens system. The matrix size of the images was 532 × 532 pixels. A hard filter was used for detection wavelength selection (700 nm).

### Statistics

For statistical analysis the software SigmaPlot™ (Systat Software Inc., San Jose, CA, USA) has been used. One-way ANOVA and Bonferroni tests have been performed for endpoint readouts while one- or two-way repeated measures ANOVA with Bonferroni tests have been used for readouts with multiple measurements. The following abbreviations were used for the indication of significance: */#: 0.01<p<0.05; **/##: 0.001≤p≤0.01 ***/###: p<0.001.

## Results

### Detection of Bleomycin-induced Lung Injury in Mice by Optimized UTE-MRI with Short Acquisition Time

**Figure 4:**
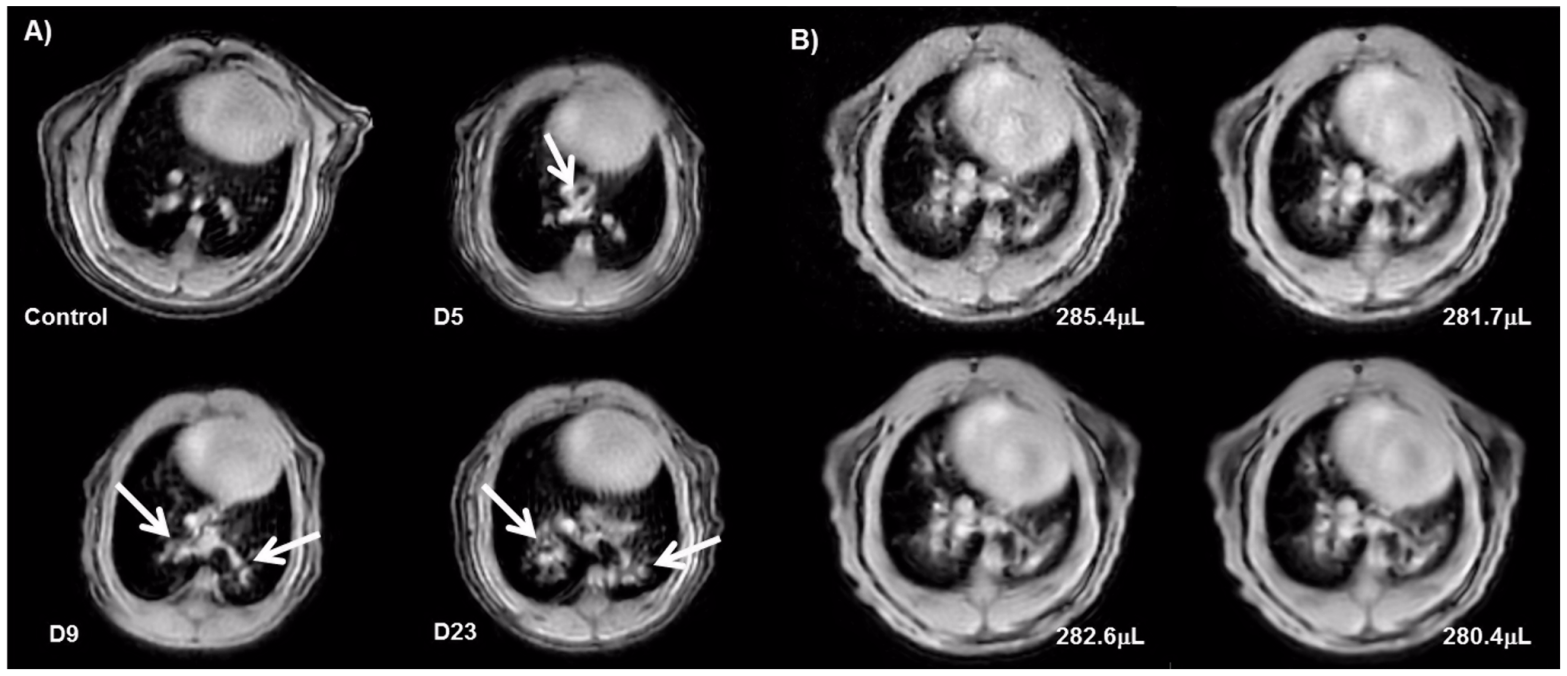
Detection of bleomycin-induced lung injury by UTE-MRI in BALB/c mice. A) Measurements (4-min-acquisition-time) on comparable slices before and at different timepoints (5, 9, and 23 days) after OA of 6 × 1.0 mg/kg bleomycin. Arrows point to bleomycin-induced lung injury. B) Comparison of UTE acquisitions with different numbers of averages in a bleomycin-treated animal. The volumes of signals in the lungs evaluated from these images were comparable for all acquisition conditions: 1 average (285.4 µL), 2 averages (281.7 µL), 4 averages (282.6 µL), 8 averages (280.4 µL). White arrows point to bleomycin induced lung injury.

Due to a reduction of the echo time and a decrease in the number of averages, the acquisition time for MRI of mice lungs could be strongly shortened as compared to previous work [25], [27]. Only four minutes were needed to generate images covering the whole lung of a mouse with ten slices, each averaged out of 2 single acquisitions (Figure 4A). The optimized acquisition was compared to longer acquisitions comprising 4 or 8 averages and resulting in data collection times of 8 and 16 minutes, respectively. Since the amount of detected lung signal, in healthy as well as in bleomycin-treated animals, was similar for all tested protocols (Figure 4B), the shorter acquisition, allowing a higher throughput and a strong reduction of narcosis time, was preferred.

### OA of Bleomycin Led to Increased Induction of Lung Fibrosis in C57BL/6 Mice Compared to IN Administration

The volume of signals induced by repeated IN bleomycin dosing (6×0.25 mg/kg) as described in Babin et al. [25] and detected by MRI never exceeded 120 µL, for different experimenters and studies performed in C57BL/6 mice. The maximum initial weight loss was of 15%. When the dose of bleomycin was doubled, namely to 6×0.5 mg/kg IN, a higher response was obtained (approximately 150 µL), but 40% of the animals had to be sacrificed prematurely due to weight loss in excess of 20%. Taken together, these preliminary data suggested that 6×0.25 mg/kg would be the best compromise for routine studies involving IN dosing of bleomycin, since fibrotic responses would be induced with low initial weight loss as shown earlier [25]. The main drawback of the IN dosing was that fibrotic lesions, despite being elicited in different lung lobes, appeared predominantly around the largest superior airways [25]. We thus aimed at verifying whether another route, namely OA, might lead to a more homogeneous distribution of fibrotic lesions throughout the lungs. As a first step, the same dose of bleomycin optimized for the IN studies, namely 6×0.25 mg/kg, has been used.

**Figure 5:**
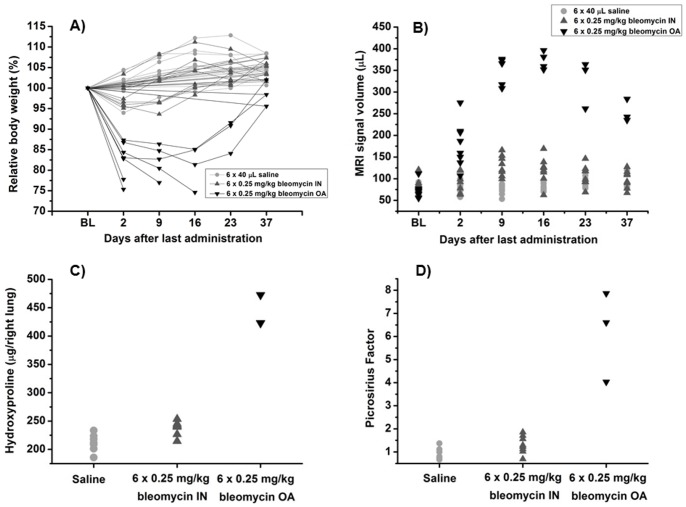
Comparison of OA to IN administration of bleomycin (6×0.25 mg/kg) in C57BL/6 mice (n = 8 per group) by *in vivo* MRI and post-mortem analyses. All data are shown as means ± STDEV. A) Relative body weight and time point of death during the study. Animals have been sacrificed directly after their last measurements. Five out of eight mice receiving OA of bleomycin had to be sacrificed prematurely due to excessive bodyweight loss (A). B) Total volume of MRI signals in the lung (µL). The signal at baseline contains contributions from vessels, whereas signals following bleomycin reflect additionally the injury inflicted by the antibiotic. C) Post-mortem determination of hydroxyproline in right lung lobes (day 37). D) Amount of collagen detected by histology of picrosirius in left lung lobes (day 37). Results are expressed as collagen content relative to mean collagen content in the lungs of saline-treated mice (picrosirius factor).

**Figure 6:**
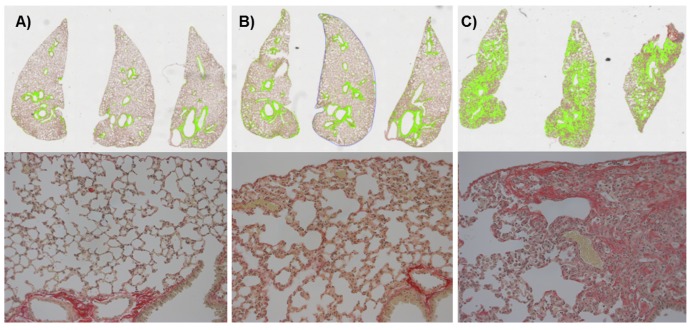
Histology of picrosirius stained lung slices from C57BL/6 mice. Collagen (green) visualized by picrosirius staining in three slices of the left lung lobe (upper row). Magnified views (×200) of a portion of the corresponding histological slices are shown in the bottom row. A) Stained collagen content in saline treated mouse lung 37 days after administration. B) Stained collagen content in a mouse lung 37 days after last IN bleomycin administration (0.25 mg/kg on six consecutive days). C) Collagen content in a mouse lung 37 days after last OA of bleomycin (0.25 mg/kg on six consecutive days). Compared to the IN administration, the amount of picrosirius was six times higher when bleomycin was oropharyngeally aspirated and the distribution of picrosirus staining was more homogeneous.

C57BL/6 mice were treated on six consecutive days with saline or bleomycin (0.25 mg/kg) by either the IN or OA route. Whereas mice dosed with bleomycin IN had initial weight loss within 10% of the body weight, five out of eight animals that received bleomycin OA had to be sacrificed prematurely because of excessive weight loss (≥ 20% of the initial body weight) (Figure 5A). MRI baseline measurements before the first administration of bleomycin allowed the use of each animal as its own control. Further MRI measurements were performed on several timepoints after last bleomycin administration. The course of MRI-detected lung injury is shown in Figure 5B. Compared to the IN route, OA administration of bleomycin led to significantly higher responses in the lungs as evidenced by MRI from a very early (day 2 after last bleomycin administration) to a late time point (day 37 after last bleomycin administration) (Figure 5B). These findings were confirmed by post-mortem analysis of the lungs (37 days after last bleomycin administration). Determination of hydroxyproline in the right lung lobe (Figure 5C) showed a significantly higher amount of hydroxyproline in lungs from mice that had received bleomycin via the OA than the IN route or the contol group (Saline). The amount of hydroxyproline in the lungs of IN-treated mice differed only slightly from the saline group. The amount of collagen in the left lung lobe was quantified by picrosirius staining in histological samples. The amount of collagen in the lungs of mice that had received bleomycin via OA was significantly higher than in all other groups (Figure 5D). There was no difference detectable between IN-treated animals and the control group. Additionally, the distribution of collagen after OA of bleomycin was more homogenous than after IN administration (Figure 6). For the OA route, fibrotic areas appeared all over the lung lobe (Figure 6C), while for the IN route, when compared to saline (Figure 6A), fibrotic lesions appeared mainly around the biggest airways (Figure 6B). No edema or emphysema has been observed by histology for either administration route. However, parenchymal infiltrate was more marked after OA of bleomycin than following IN administration of the antibiotic.

**Figure 7:**
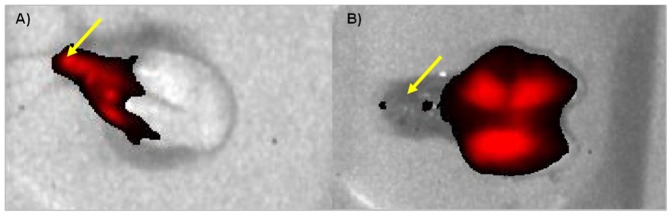
Ventral NIRF images of the lungs from two mice 60 min after instillation of the dye Cy5.5 (0.1 mg/mL) either via the IN route (A) or via OA (B).Yellow arrows point to the trachea.

For comparison, the distribution of Cy5.5 for both administration routes has been studied on isolated lungs from a separate cohort of animals using NIRF imaging. Sixty minutes after OA of the dye, a homogenous distribution of the fluorescence signal all over the lung was observed. After IN administration of the dye, at the same timepoint, the fluorescence signal appeared mainly in the upper part of the lung and the trachea (Figure 7).

**Figure 8:**
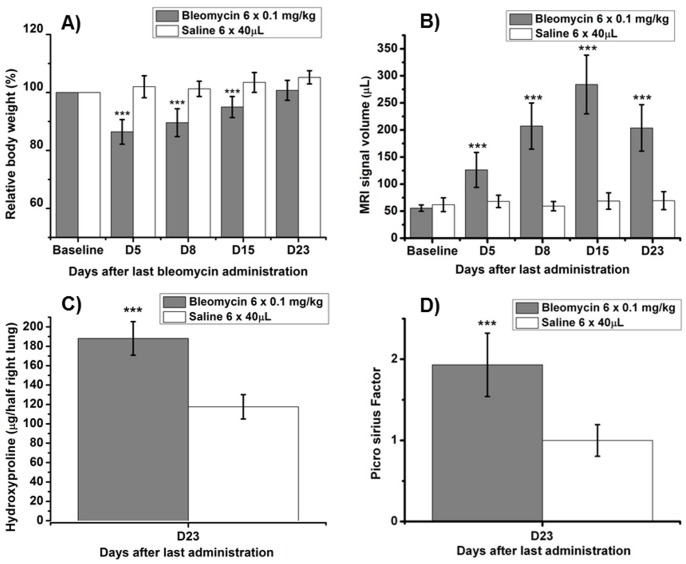
Detection of bleomycin-induced lung injury in C57BL/6 mice by UTE-MRI, picrosirius staining, and hydroxyproline determination. Animals were treated with 0.1 mg/kg bleomycin (n = 9) or vehicle (n = 5) on six consecutive days via OA. Data are shown as means ± STDEV. A) Relative body weight during the course of the experiment. The statistical significance values ***p<0.001 represent comparisons between saline and bleomycin-treated mice, at each time point. B) Total volume of MRI signals in the lung (µL). The signal at baseline contains contributions from vessels, whereas signals following bleomycin administration reflect additionally the injury induced by the antibiotic. The statistical significance values ***p<0.001 indicate comparison to baseline. C) Post-mortem determination of hydroxyproline in right lung lobes D) Picrosirius factor expressing the amount of collagen relative to the mean collagen content assessed in the lungs of saline-treated mice. The statistical significance value ***p<0.001 means comparison to saline treatment.

A known negative side effect of bleomycin-induced lung fibrosis in small rodents is a prominent loss of body weight. After OA of bleomycin (6 × 0.25 mg/kg), not only the amount of induced lung fibrosis was higher than for IN administration, but also the body weight loss (Figure 5A). For ethical reasons, five out of eight mice had to be sacrificed due to excessive weight loss (∼20% of the initial body weight). Thus, for the OA of bleomycin a dose reduction was required. While the initial weight loss was tolerable (<20%) (Figure 8A), an adequate response has been obtained with six times 0.1 mg/kg OA of bleomycin (Figure 8B–D).

### Dose Adaptation of OA Administered Bleomycin in BALB/c Mice

**Figure 9:**
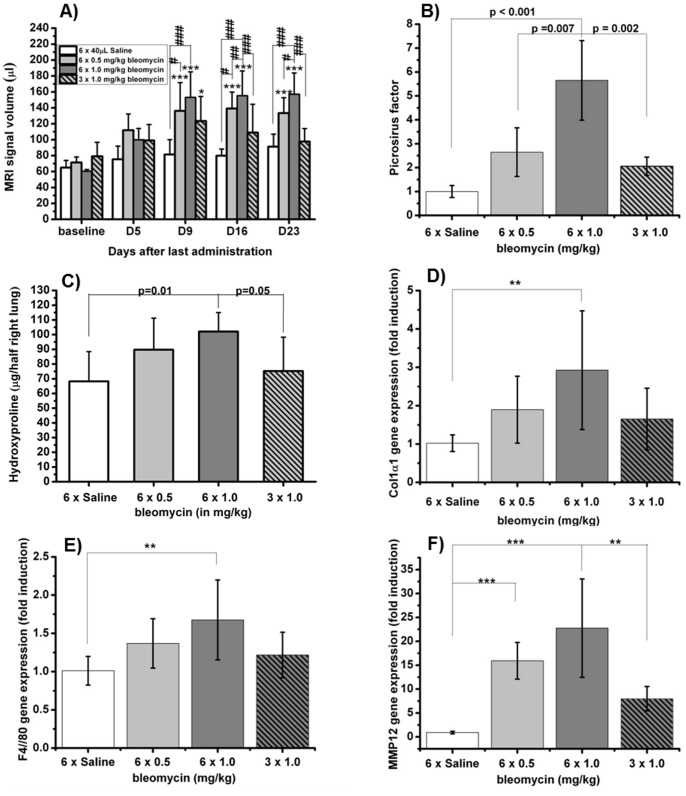
Dose adaptation of bleomycin to OA route and comparison of different bleomycin doses in BALB/c mice by UTE-MRI and post-mortem analyses (n = 8 mice for each group except n = 4 mice for histological analysis). Data are shown as means±STDEV. One-way ANOVA (Two-Way repeated measures ANOVA for MRI-results) with Bonferroni tests was used for statistical analysis. A) Total volume of MRI signals detected in the lung by UTE-MRI The signal at baseline contains contributions from vessels, whereas signals following bleomycin administration reflect additionally the injury induced by the antibiotic. B) Collagen content in left lung lobes detected by picrosirius staining. The picrosirius factor expresses the amount of collagen relative to the mean collagen content in the lungs of saline-treated mice. B) Post-mortem determination of hydroxyproline in right lung lobes (day 23); Data are shown as Mean ± STDEV of µg hydroxyproline per right lung. D-F) mRNA expression level of Col1α1, F4/80, and MMP12 compared to expression of HPRT. Data shown as means±STDEV of fold induction compared to control group.

The motivation to analyze the effects of OA of bleomycin in BALB/c mice was the fact that many transgenic models have a BALB/c background. Former studies have shown that a much higher dose of bleomycin is needed to induce an amount of lung fibrosis in BALB/c mice that is comparable to the response in C57BL/6 mice [25], [45], [46]. Thus, an up to ten times higher dose of bleomycin was expected to induce an adequate amount of lung fibrosis in BALB/c mice when using the OA method. Three different doses of bleomycin were tested in groups of eight animals and compared to saline-treated mice. The induced lung injury was detected by UTE-MRI while picrosirius staining as well as determination of hydroxyproline and RT-PCR were performed post-mortem. Compared to baseline, lung injury detected by UTE-MRI was significantly increased in animals treated with either 0.5 or 1.0 mg/kg bleomycin on six consecutive days (Figure 9A). Mice that received bleomycin that was divided between only three days (3 × 1.0 mg/kg, every second day) demonstrated a significant increase of MRI lung signal during the initial phase only (up to nine days after last administration). A treatment with 1.0 mg/kg on six consecutive days induced the significantly highest volume of lung injury in the four compared groups as evidenced by MRI and led to a tolerable loss of bodyweight (≤15%; data not shown). Regarding post-mortem analyses (23 days after last bleomycin administration), only a sixfold administration of 1.0 mg/kg bleomycin induced an amount of lung fibrosis that led to a significant higher amount of collagen as detected by picrosirius staining (Figure 9B), hydroxyproline (Figure 9C), and Col1α1 gene expression (Figure 9D). The expression of MMP12 and F4/80, both markers for the presence of tissue macrophages [47]–[51], was significantly increased after a sixfold administration of 1.0 mg/kg bleomycin (Figure 9E–F). Neither edema nor emphysema has been observed histologically. Administration of 6 × 0.5 and 3 × 1.0 mg/kg bleomycin led to moderate, 6 × 1.0 mg/kg bleomycin to marked parenchymal cellular infiltration. The correlations between MRI, and all other readouts (picrosirius, hydroxyproline, and gene expression of col1α1, F4/80 and MMP12; Table 1) were significant (p at least 0.005). The strongest correlation (R = 0.90, p<0.00001) was found between the volume of MRI lung signal and the amount of collagen (picrosirius staining).

**Table 1:** Correlations between MRI and all other readouts on day 23 analyzed in the present study for detection of bleomycin-induced lung fibrosis in BALB/c mice (n = 32; except for Picrosirius n = 16): total MRI signal volume, picrosirius staining, hydroxyproline, and gene expression of Col1α1, F4/80 and MMP12.

Correlation between MRI and	R	p
Picrosirius	0.90	<0.00001
F4/80 gene expression	0.68	<0.0001
MMP12 gene expression	0.60	<0.001
Col1α1 gene expression	0.60	<0.001
Hydroxyproline	0.48	0.005

Saline-treated BALB/c animals (n = 8, n = 4 for picrosirius) were used as controls.

### OA Administration in Rats

**Figure 10:**
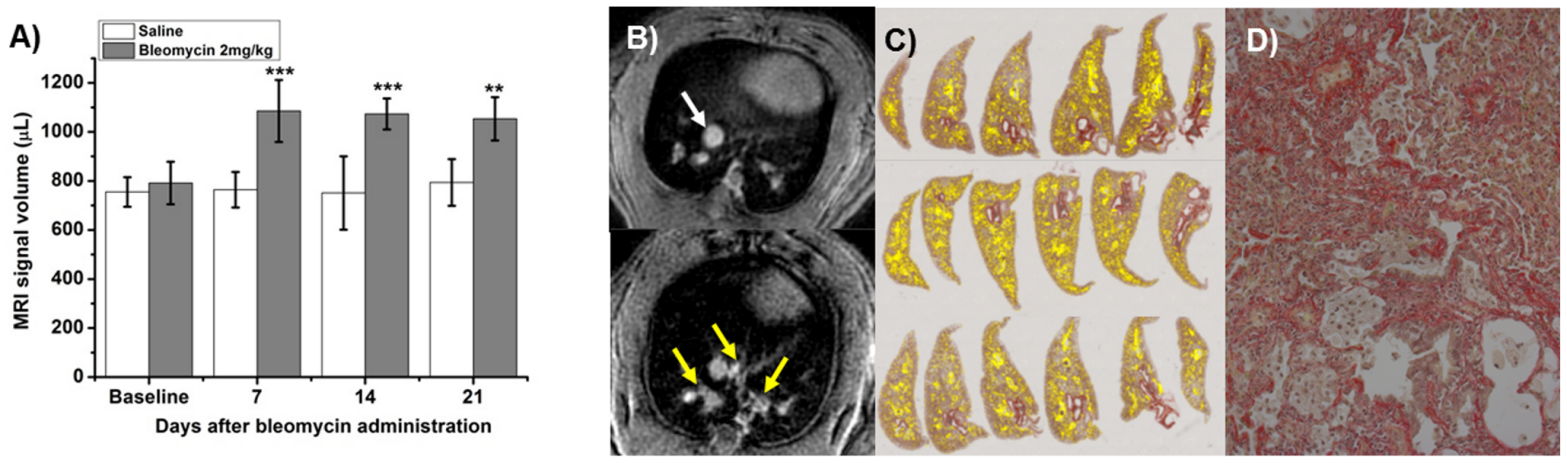
Oropharyngeal aspiration of 2 mg/kg bleomycin or of saline in Sprague Dawley rats (n = 5 per group); A) Volumes of signals (means±STDEV) detected by UTE-MRI in the lungs of rats before and after bleomycin; Two-way repeated measures ANOVA with Bonferroni tests was performed. The statistical significance values ***p<0.001, **p<0.01 indicate comparison to baseline as well as comparison to Saline group. B) Signals at baseline contain contributions from vessel. (white arrow in upper panel), whereas signals following bleomycin administration reflect additionally the injury induced by the antibiotic (yellow arrows in lower panel). C) Histological analysis of the left lung lobes. Yellow areas are representing collagen, stained by picrosirius; neither emphysema nor edema was observed. D) Magnified view (×200) of one of the histological slices demonstrating widespread fibrosis as evidenced by picrosirius staining.

Currently, IT is the most often used route to apply substances into rat lungs. It is a well-established method and may still be the technique of choice for single dosing, but especially for multiple dosing there might be a preference for a less invasive administration route as for instance OA. Since there are not many reports available that describe the use of OA in rats, the feasibility of this administration route has been first tested in a single animal. One hundred microliters of saline were administered via OA, as described before. The liquid was rapidly aspired and no interruption of breathing, which is a common complication during IT administration, occurred. After a single dose of bleomycin (2 mg/kg) via OA, the development of lung injury was detected by UTE-MRI at different time points. Figure 10A shows a significant increase of MRI lung signal already seven days after administration that stayed constant until 21 days after bleomycin administration. A homogenous collagen accumulation all over the lung with comparable intensity was detected by picrosirius staining in the left lung lobes (Figure 10C).

## Discussion

Despite substantial research during the last years, currently no efficacious treatment exists for lung fibrosis. The availability of a robust animal model allowing reliable predictions is important for the development of therapeutics against lung fibrosis. Fibrosis can be modeled in mice by the instillation of bleomycin into the lung [15], [52]. Having access to non-invasive imaging is an advantage in the context of therapeutic treatment analysis, as randomization before treatment is feasible and the effect of a therapy on each single animal can be followed longitudinally. This is especially important in view of the variability of the responses induced by bleomycin. For instance, the bleomycin-induced injury can be quantified at a certain time point, just before beginning of treatment. The lung injury quantified non-invasively would then serve as baseline, immediately prior to treatment initiation. Alternatively, the responses induced by bleomycin and quantified by MRI can be used to randomize the treatment groups, in order to have similar mean values at the beginning of treatment across the different groups of animals.

Induction of lung fibrosis by IN or IT administered bleomycin has been used to investigate the effect of potential therapeutics or the influence of genes on the development of lung fibrosis for target finding [53], [54]. Since, especially in mice, IT administration is difficult to perform and IN administration might lead to inhomogenous responses, because of its dependence on different parameters as depth of anesthesia or animal position [19], it seemed to be appropriate to investigate the potential of the OA technique for the described model.

In the present work we demonstrated that the OA administration route offers a possibility to further optimize the murine bleomycin model. Intra-nasal administration and OA of bleomycin were compared in C57BL/6 mice regarding the amount (detected by UTE-MRI, histology, and hydroxyproline determination) and the distribution of induced lung injury. The volumes of administered fluid were chosen based on earlier work [19], [20], [22]. Using autoradiography Southam et al. [19]have shown that IN administration of 25 µl of fluid to mice leads to a similar uptake in the lung than 40 µl. Conversely, IN administration of lower volumes, e.g. 5 µl, leads to a preferential uptake in the brain. In our hands, we verified that it was easier to administer a volume of 25 µl than of 40 µl, as the latter volume significantly favored the formation of bubbles, resulting in extravasation of fluid out of the nasal cavity. On the other hand, using scintigraphy Foster et al. [20] demonstrated that OA of 50 µl of fluid led to a significantly better uptake in the lungs than 25 µl. We followed the paper of Lakatos et al. [22] and adopted 40 µl for OA delivery. Bleomycin administration via the OA route induced significantly higher amounts of lung injury, detected by MRI *in vivo* and identified as fibrosis by histology, as compared to IN administration. One explanation for this observation might be the obviation of swallowing due to pulling out the tongue during the OA, whereas the animals are, theroretically, still able to swallow during IN administration. A second explanation is proposed by the study of Southam et al., who investigated the distribution of IN administered ^99m^Tc-labelled sulfide-colloid into different tissues [19]. When 25 µL of ^99m^Tc-labelled sulfide-colloid was administered IN, only 40–50% reached the lung. The rest of the substance could be found in the brain (about 40%) and stomach (5–10%). According to our knowledge, no such study has been performed for the OA route but it might be suggested that, during OA of liquids, less material reaches the brain since the substance does not directly pass the nasal cavity with its access to the brain. Thus, the main part of oropharyngeally aspirated liquid should reach the lung. The more pronounced response induced by the OA of bleomycin allowed a significant dose reduction from a total of 1.5 mg/kg to 0.6 mg/kg in C57BL/6 mice. This means a model refinement not only due the dose reduction of bleomycin but also because of the accompanying less pronounced body weight loss. Of note, the response induced by IN administration of bleomycin here was lower than that attained earlier for the same model [25]. A possible explanation could be the different suppliers of bleomycin in both studies.

The dose of bleomycin in BALB/c mice could be successfully adapted to OA. A six fold dosing of 1.0 mg/kg bleomycin induced an adequate increase of MRI lung signal which is strongly correlating with lung collagen content (picrosirius), collagen gene expression (Col1α1), hydroxyproline content, and markers for the presence of tissues macrophages that are supposed to be involved in fibrotic processes. The amount of detected MRI signal was of the same order of magnitude as the volume of lung injury in C57BL/6 mice (compare Figures 8B and 9A), and induced a tolerable initial body weight loss of less than 20%. In other words, in order to obtain a similar response in male BALB/c mice as that obtained in male C57BL/6 mice, the total bleomycin dose had to be increased by a factor of 10. This is consistent with previous results obtained using repeated IN administrations of bleomycin, showing that the responses induced by the same total dose of the antibiotic were approximately 10 times weaker in male BALB/c compared to male C57BL/6 mice [25]. Additionally to dose adaptation, it was tested in BALB/c mice whether the number of bleomycin administrations could be reduced from six to three. A threefold dose of 1.0 mg/kg bleomycin did not induce an adequate response as indicated by several readouts (Figure 9). A threefold dose of 2.0 mg/kg bleomycin (identical total dose as the preferred 6 × 1.0 mg/kg) was tested but excluded since the higher single dose evoked breathing interruptions. Therefore, a lower number of bleomycin administrations decreased the amount of induced lung fibrosis and is thus not recommended.

In addition to dose reduction, changing from IN administration to OA of bleomycin evoked another improvement of the lung fibrosis model. There was a clear change apparent regarding localization of fibrotic lesions. Picrosirius staining (Figures 6C and 10C) showed a homogenous distribution of fibrotic lesions after OA in comparison to IN administration of bleomycin (Figure 6B). These different localizations of induced fibrosis can be explained by NIRF images of OA or IN administered Cy5.5 (Figure 7). Whereas 60 minutes after OA of Cy5.5 the fluorescence signal could be detected all over the lung lobes (Figure 7B), the IN administered dye was localized in the trachea and the upper part of lung (Figure 7A). Assuming that bleomycin is distributed in a comparable manner, this would explain why after IN administration of bleomycin the fibrotic lesions were primarily found around the main airways whereas the OA of bleomycin also induced fibrosis in the periphery of the lung. To have fibrotic areas in the lung periphery might be an important characteristic of a lung fibrosis model since this means a better comparability to the human disease where the deposition of collagen occurs with a peripheral distribution [55].

A similar distribution of fibrotic lesions could be found in rat lungs after OA of bleomycin. Since OA is not a commonly used method for lung instillation in rats, the feasibility was first tested in a small number of animals. The method was found to be easy, fast to apply and effective, since the amount of bleomycin that induced fibrosis was comparable to the amount in IT-treated rats [17]. Thus, OA in rats should be taken into account when a less invasive alternative to IT administration is needed, for instance for multiple dosing.

The UTE acquisition sequence for *in vivo* imaging has been optimized here for increased throughput in view of pharmacological applications. Compared to earlier work [25], [27], the acquisition time of MR images of mice lungs could be considerably reduced by the use of the UTE sequence with short echo time. Although the method provides only a global view of the injury induced by bleomycin, the strong correlation (R = 0.90, p<0.00001) between the signals quantified by MRI and the amount of collagen detected histologically (picrosirius staining) indicates that MRI reflected the development of fibrosis in the model. Moreover, although less strong, there was also a significant correlation between the MRI signals and the hydroxyproline content (R = 0.48, p = 0.005) as well as the expression of genes that are suggested to be involved in fibrotic processes. The expression of Col1a1, the gene that is encoding for the major component of type 1 collagen, but also the macrophage-specific expression of MMP12 and F4/80 47–51 were highly correlated (p<0.001) with the MRI signals.

In summary, repeated OA administration of bleomycin led to sustained fibrosis in mice, with an acceptable initial weight loss of less than 15%. The method was easy to perform, fast and effective, as the total dose of bleomycin could be reduced compared to IN dosing. Moreover, a more homogeneous distribution of fibrotic lesions has been obtained than when using IN dosing. In rats, the OA route could be taken into consideration for repeated dosing.
